# Volatile Signals From Guava Plants Prime Defense Signaling and Increase Jasmonate-Dependent Herbivore Resistance in Neighboring Citrus Plants

**DOI:** 10.3389/fpls.2022.833562

**Published:** 2022-03-10

**Authors:** Siquan Ling, Syed Arif Hussain Rizvi, Ting Xiong, Jiali Liu, Yanping Gu, Siwei Wang, Xinnian Zeng

**Affiliations:** ^1^Guangdong Engineering Research Center for Insect Behavior Regulation, College of Plant Protection, South China Agricultural University, Guangzhou, China; ^2^Insect Pest Management Program, National Agricultural Research Centre, Islamabad, Pakistan; ^3^Plant Protection Research Institute, Guangdong Academy of Agricultural Sciences, Guangzhou, China

**Keywords:** coexistence, volatile organic compounds, eavesdropping, defense response, JA signaling, guava, citrus, *Diaphorina citri*

## Abstract

Intercropping can reduce agricultural pest incidence and represents an important sustainable alternative to conventional pest control methods. Citrus intercropped with guava (*Psidium guajava* L.) has a lower incidence of Asian citrus psyllid (ACP, *Diaphorina citri* Kuwayama) and huanglongbing disease (HLB), but the mechanisms are still unknown. In this study, we tested whether volatile organic compounds (VOCs) emitted by guava plants play a role in plant–plant communications and trigger defense responses in sweet orange (*Citrus sinensis* L. Osbeck) in the laboratory. The results showed that the behavioral preference and developmental performance of ACP on citrus plants that were exposed to guava VOCs were suppressed. The expression of defense-related pathways involved in early signaling, jasmonate (JA) biosynthesis, protease inhibitor (PI), terpenoid, phenylpropanoid, and flavonoid biosynthesis was induced in guava VOC-exposed citrus plants. Headspace analysis revealed that guava plants constitutively emit high levels of (*E*)-β-caryophyllene and (*E*)-4,8-dimethyl-1,3,7-nonatriene (DMNT), which can induce the accumulation of JA and promote stronger defense responses of citrus to ACP feeding. In addition, exposure to guava VOCs also increased the indirect defense of citrus by attracting the parasitic wasp *Tamarixia radiata*. Together, our findings indicate that citrus plants can eavesdrop on the VOC cues emitted by neighboring intact guava plants to boost their JA-dependent anti-herbivore activities. The knowledge gained from this study will provide mechanisms underlying citrus-guava intercropping for the ecological management of insect pests.

## Introduction

Volatile organic compounds (VOCs), which are continuously produced and emitted by plants into their surroundings, are the crucial signals in long-distance plant-plant communications (Ninkovic et al., 2016). Plants have developed complex mechanisms to detect, perceive, and respond to fluctuating VOCs from neighboring plants, making plant-plant interactions *via* a continuous and dynamic process (Pierik et al., 2013; Ninkovic et al., 2021; Sharifi and Ryu, 2021). In many cases, undamaged plants can eavesdrop on VOCs emitted by a neighboring plant under biotic stresses (e.g., herbivore- and pathogen-attacked) and react by activating or priming defense responses (Underwood et al., 2014; Takabayashi and Shiojiri, 2019). Meanwhile, some plant species can emit high levels of VOCs, such as aromatic compounds and sesquiterpenes, even when they are not damaged or stressed (Sukegawa et al., 2018; Huang et al., 2019). These constitutively emitted VOCs can also induce or prime defense responses in neighboring plants, as demonstrated in volatile chemical interactions between weed species and barley (Glinwood et al., 2004; Ninkovic et al., 2009; Dahlin and Ninkovic, 2013), onion and potato (Ninkovic et al., 2013; Vucetic et al., 2014; Dahlin et al., 2015), mint varieties and soybean (Sukegawa et al., 2018), and molasses grass and maize (Tolosa et al., 2019). The interactions resulted in higher VOC emissions in receiving plants, which are involved in direct defense (directly affecting an herbivore’s physiology or behavior) and indirect defense (enhancing the effectiveness of natural enemies of the herbivore) of plants against herbivorous insects (Vucetic et al., 2014; Tolosa et al., 2019). This study revealed a new mechanism that affects insect pests in intercropping systems. However, these effects only occur in specific combinations of plant species, indicating that the adaptive strategy of the plants exposed to VOCs depends strongly on the emitter’s identity and physiological status (Ninkovic et al., 2016; Kigathi et al., 2019).

Guava, *Psidium guajava* L. (Myrtaceae), which is widely cultivated in many tropical and subtropical countries, is valued for its characteristics, such as flavor, aroma, and nutritional value of its fruit, in addition to being a potential source of phytochemicals (de Souza et al., 2018). *Citrus* (Rutaceae) is also a widely cultivated fruit crop. Intercropping is a commonly used tactic for indirect pest management, particularly in diverse agroecosystems typical of the tropics. It has been revealed that citrus intercropped with guava has a lower incidence of huanglongbing (HLB) disease and a lower infestation level of Asian citrus psyllid (ACP; *Diaphorina citri* Kuwayama), the vector of HLB bacteria (Gottwald et al., 2014). Some guava VOCs can play a role in repelling or interfering with ACP, thereby limiting the spread of HLB (Zaka et al., 2010; Onagbola et al., 2011; Silva et al., 2016; Alquézar et al., 2017). Barman et al. (2016) indicated that citrus branch exposure to guava branch VOCs can rapidly alter citrus olfactory cues, reducing the attractiveness of citrus to ACP. This result may further reveal the role of citrus-guava intercropping in the regulation of the ACP population. Whether and how guava VOCs boost defense responses in eavesdropping citrus plants, however, is unknown.

This study focused on the effects of guava VOC(s) on the levels of herbivore resistance, defense-related gene expression, phytohormones, and volatile and non-volatile secondary metabolites of citrus plants. The electrophysiology and olfactory responses of ACP and its natural enemy to the induced VOCs of citrus were also examined. The knowledge gained in this study will provide molecular mechanisms underlying how eavesdropping plants respond to VOC signals from guava plants, as well as reveal the possibility of using guava VOC(s) as defense inducers in citrus orchards.

## Materials and Methods

### Plants and Insects

Sweet orange [*Citrus sinensis* (L.) Osbeck cv. Hongjiang] and guava (*P. guajava* L. cv. Yanzhi) plants (1.5-years old, 40−50 cm tall), which were obtained from the College of Horticulture, South China Agricultural University (SCAU), Guangzhou, China, were grown in pots (diameter: 10 cm, height: 16 cm) and separately cultured in greenhouses more than 3 months before use (22−28°C, 70−80% RH, 16/8 h photoperiod with 300 μmol⋅m^–2^⋅s^–1^ supplemental lighting at the canopy level). The selected citrus plants had been lightly pruned earlier so that numerous flushing shoots were present at the time of testing.

Asian citrus psyllid (*D. citri*) was continuously reared on citrus (sweet orange) in incubators (26 ± 1°C, 70−80% RH with a 16/8 light/dark photoperiod). *Tamarixia radiata* Waterston, a parasitic wasp of ACP, was collected from naturally infested orange trees in SCAU and reared on an ACP-citrus system in incubators (Liu et al., 2019). Before bioassays, mummified nymphs of ACP, which were parasitized by *T. radiata*, were introduced into Petri dishes (12 cm) under laboratory conditions. After *T. radiata* adults emerged, they were collected and reared in a glass tube with a 20% sugar solution. Usually, *T. radiata* can finish their mating behavior during the first 2 days following emergence. The mated female wasps were segregated into 1-ml centrifuge tubes before use.

### Exposure of Citrus Plants to Guava Volatile Organic Compounds

Exposure of citrus plants to guava VOCs was conducted in glass cages (60 × 60 × 80 cm) with double-pass valves (Supplementary Figure 1A). A single guava plant was placed next to the citrus plant (10 cm apart) in a glass cage under laboratory conditions (26 ± 1°C, 70−80% RH with a 16/8 light/dark photoperiod). Only a citrus plant in the cage was set as a control. Purified and humid air was pumped into the chamber at 1 L min^–1^ and then pumped outside of the house. Before the end of the 14-day co-cultivation, half of the citrus was infested by 100 ACP adults (sex ratio = 1:1) for 24 h. Afterward, guava VOC-exposed, ACP-infested, guava + ACP-treated, and control groups were obtained. In addition, a citrus neighbor (CS-neighbor) instead of the guava neighbor was also used as a negative control in the behavioral preference bioassay. The sender plants and insects were removed, and the treated citrus plants were used for preference bioassay, volatile collection, or direct freezing in liquid nitrogen and stored at −80°C for gene expression, untargeted metabolomics, and phytohormone analysis.

### Insect Preference and Performance Experiments

Responses of insects to the odor of citrus were assessed using Y-tubes (Barman et al., 2016) and free choice behavioral experiments (George et al., 2019). Y-tube arms were connected to 5-L Polytetrafluoroethylene (PTFE) bags containing treatment and control citrus with airflow of 200 ml min^–1^. A 10-W light was placed directly in front of the Y-tube to drive the insects forward. A single ACP or female wasp was released and observed within 8 min. Insects were chosen if they moved a minimum of 1/3 of the arm away from the junction. Each tested insect was used only once. Combined responses of 30 ACP or 10 female wasps were used as the replicates. Between replicates, positions of the odor sources were exchanged to avoid directional bias, and the Y-tube was washed and dried. In the free-choice experiment, thirty ACP adults (∼1:1 male:female ratio) were introduced into each screen cage (60 cm × 60 cm × 80 cm) containing two citrus plants (one guava VOC-exposed and one non-exposed), and the number of ACP adults on each plant was carefully recorded at 24 h after introduction.

The oviposition of ACP female wasps and subsequent performance of next-generation on guava VOC-exposed citrus plants were demonstrated (George et al., 2019). Citrus with tiny buds was exposed to guava VOCs for 14 days, and then, three tender shoots (2−3 cm) were kept for oviposition. In a no-choice test, a guava VOC-exposed citrus plant was introduced into the screen cage, and the control plant was placed in a separate cage, while the guava VOC-exposed and control citrus plants were placed into the same oviposition cage in the free-choice test. Six 10- to 15-day-old adults of ACP (sex ratio = 1:1) were placed in each cage and were allowed to oviposit for 48 h. Then, the adults were removed, and the eggs were photographed using a microscope. In the no-choice tests, the plants with eggs were continuously cultivated. The number of nymphs and their nymphal instars were recorded 10 days after the introduction of female wasps. Cages were checked for adult emergence, and new adults were recorded daily and removed. Insect preference and performance measurements were conducted under laboratory conditions (26 ± 1°C, 70−80% RH with a 16/8 light/dark photoperiod). Experiments were replicated five times.

### RNA Preparation and RNA-Seq Analysis

Total RNA was extracted from each leaf sample using the RNAprep Pure Plant Kit (TIANGEN, Beijing, China), following the manufacturer’s protocol. RNA purity and integrity were determined using a NanoDrop 2000 spectrophotometer (Thermo Fisher Scientific, Waltham, MA, United States) and Bioanalyzer 2100 (Agilent Technologies, Santa Clara, CA, United States). The libraries were constructed using the TruSeq Stranded mRNA LT Sample Prep Kit (Illumina, San Diego, CA, United States), according to the manufacturer’s protocol. RNA sequencing was carried out on Illumina NovaSeq 6000 platform (OE Biotech Co., Ltd., Shanghai, China). Three independent biological replicates were used for each treatment. A total of 12 libraries were obtained and deposited in the Sequence Read Archive of NCBI (accession number: PRJNA778232). A total of 81.23 G of clean data and 6.51−7.15 G of clean data per library were generated after filtering the raw data. The Q30 base was distributed in 92.17−93.0% and the average guanine and cytosine content was 44.76%. After *de novo* assembly using the Trinity package (Grabherr et al., 2013), a total of 35,039 unigenes were obtained with a total length of 47,714,801 bp and an average length of 1,361.76 bp. Data were generated and basic local alignment search tool (BLAST) aligned with public databases [non-redundant protein sequence database (NR), Swiss-Prot, Kyoto encyclopedia of genes and genomes (KEGG), clusters of orthologous groups for eukaryotic complete genomes (KOG), evolutionary genealogy of genes: non-supervised orthologous groups (eggNOG), gene ontology (GO), and Pfam]. A total of 26,383 (75.30%) unigenes were annotated to the NR database. RNA-seq data were normalized to fragments per kilobase per million mapped reads (FPKM) values to quantify gene expression levels. The differentially expressed genes (DEGs) between various samples were identified and filtered using estimateSizeFactors and nbinomTest functions of the DESeq R package (Anders and Huber, 2012). We used a *P*-value of <0.05 and log_2_∣fold change∣ > 1 as thresholds to define DEGs.

### Quantitative Real-Time PCR

Total RNA was extracted with the same method as for RNA-seq analysis. cDNA was synthesized from 1 μg of total RNA using the TaKaRa PrimeScript™ RT reagent kit. qRT-PCR analysis was performed using a Bio-Rad CFX96 Real-Time PCR system (Bio-Rad, Hercules, CA, United States) with the SYBR Green Premix Pro Taq HS qPCR Kit (Accurate Biotechnology, Changsha, China) in a 20-μl reaction volume under the following conditions: 95°C for 30 s, 40 cycles of 95°C for 15 s, and 60°C for 30 s, and a final melting stage from 60 to 95°C. Specific primers are listed in Supplementary Table 1. The primer specificity was monitored using 2% agarose gel electrophoresis and melting curve analysis. Amplification efficiency in qPCR was confirmed (90−110%) from a standard curve with serial dilutions (5- or 10-fold gradient dilution) of the cDNA template. The 2^–ΔΔCt^ method was used to calculate relative expression levels, and data were normalized against reference genes *FBOX* and *UPL7*, which are widely used as reference genes from citrus (Mafra et al., 2012).

### Phytohormone Quantification

Phytohormones, including ABA, SA, JA, and JA-Ile, were analyzed according to previous studies (Forcat et al., 2008; Zhang et al., 2020) with modifications. A total of 50 mg of leaf powder was extracted with 1 ml of ice-cold 10% methanol/water (v/v) containing 0.125% acetic acid on ice for 30 min, centrifuged at 12,000 rpm for 10 min (4°C), and then filtered through 0.22-μm microfilters. Five independent biological replicates were used for each treatment. Samples were analyzed using an Agilent 1200 HPLC system (Agilent Technologies, Santa Clara, CA, United States) coupled to an AB Sciex Triple Quad 4000 mass spectrometer (AB Sciex, Framingham, MA, United States), and 10 μl of each sample was injected onto an Agilent Poroshell EC-C18 column (150 mm × 3.0 mm, 2.7 μm; Agilent Technologies, Santa Clara, CA, United States) with a flow rate of 400 μl min^–1^ at 35°C. The mobile phases were 0.1% formic acid in water (A) and acetonitrile (B). Separation was performed using an isocratic elution consisting of 30% A and 70% B over 5 min. The eluent was introduced into the electrospray ionization source of a tandem mass spectrometer operating with the following settings: negative mode, ion source temperature (450°C), ion source gas (60 psi), capillary voltage (−4,500 V), declustering potential, and collision energy (ABA: −65 V and −16 eV; SA: −52 V and −24.3 eV; JA: −61 V and −24.7 eV; JA-Ile: −92 V and −28.3 eV). Compounds were quantified in multiple reaction monitoring modes (MRM) using calibration curves obtained by plotting the concentration (0.2, 0.5, 1, 2, 5, 10, and 20 ng ml^–1^) and the peak area of quantitative ion for each standard. The mass transitions of MRM were as follows: ABA: 263.1 > 152.9; SA: 136.8 > 93.2; JA: 209.0 > 59.1; and JA-Ile: 322.6 > 130.1.

### Untargeted Metabolomic Analysis

Semipolar metabolite analysis was conducted according to Jia et al. (2019), with some modifications. A total of 80 mg of leaf powder was extracted with 1 ml of methanol/water (7/3, v/v) containing 0.125% acetic acid, and 20 μl of 2-chloro-l-phenylalanine (0.3 mg ml^–1^ in methanol) was added as an internal standard. The supernatants were filtered through 0.22-μm microfilters. Six independent biological replicates were used for each treatment. The extracted samples were analyzed using an Acquity UHPLC (Waters Corporation, Milford, MA United States) coupled with a Triple TOF 5600 system (AB Sciex, Framingham, MA, United States) in both ESI positive and ESI negative ion modes. At 45°C, 2 μl of each sample were injected onto an Acquity UPLC BEH C18 column (1.7 μm, 2.1 mm × 100 mm) with a flow rate of 400 μl min^–1^. The binary gradient elution system consisted of (A) water (containing 0.1% formic acid, v/v) and (B) acetonitrile/methanol (2/3, v/v, containing 0.1% formic acid), and separation was achieved using the following gradient: 0 min, 5% B; 2 min, 25% B; 9 min, 100% B; 13 min, 100% B; 13.1 min, 5% B and 16 min, 5% B. Data acquisition was performed in full scan mode combined with the information-dependent acquisition mode (m/z range: 50–1,000). The parameters were set as follows: ion spray voltages, 5,500 V (+) and −4,500 V (−); ion source temperature, 550 °C; collision energies, 10 eV (+) and −10 eV (−); curtain gas, 35 PSI; interface heater temperature, 550 °C. Data files generated from the liquid chromatography (LC)-mass spectroscopy (MS) platform were processed using progenesis QI (version 2.3) software (Waters Corporation, Milford, MA United States) for baseline correction, mass spectra extraction, and mass signal alignment. Data from positive and negative ion modes were combined. Metabolites were putatively annotated based on public databases [Human Metabolome Database (HMDB) and Lipid Metabolites and Pathways Strategy (LIPID MAPS)] and a self-built database (Lu-Ming Biotech Co., Ltd., Shanghai, China). The metabolite diversities were visualized as alterations by principal component analysis (PCA) and (orthogonal) partial least squares-discriminant analysis [(O)PLS-DA]. Variable importance in the projection (VIP) >1 and a *P* < 0.05 (two-tailed Student’s *t*-test) were considered differentially expressed metabolites.

### Volatile Organic Compound Extraction and Analysis

Plant parts were stored in 5-L Teflon bags. Before sampling, the clean air was flushed through the collection system for 15 min to remove the VOCs produced during equipment installation. Headspace VOCs were adsorbed for 6 h (10:00–16:00) on a HayeSep Q adsorbent (50 mg, 80/100 mesh, Supelco, Bellefonte, United States) using charcoal-filtered air at a rate of 400 ml min^–1^. After collection, the traps were extracted with 500 μl of hexane containing nonyl acetate (1.75 ng μl^–1^) as the internal standard and concentrated to 100 μl under a soft stream of nitrogen. In this study, VOCs from citrus branches, guava branches, and guava roots in soil were collected. The VOCs from the soil were set as the background of roots. Each plant was considered an independent biological replicate, and six replicates were used for each treatment. Samples were analyzed using an Agilent 7890B GC-5977A MSD or 8890 GC-5977B MSD equipped with an HP-5MS column (30 m × 0.25 mm of inner diameter and 0.25 μm of film thickness, Agilent Technologies, Santa Clara, CA, United States). In spitless mode, 1 μl of the sample was injected (helium, 1.0 ml min^–1^). The injector temperature was set to 250°C, and the oven temperature programs were as follows: 40°C (1-min hold), 8°C min^–1^ to 200°C (1-min hold), and 20°C min^–1^ to 280°C (5-min hold). VOCs were identified based on their RIs (calculated using C7−C30), by comparison of their mass spectra with those stored in the NIST library and were performed by coinjection of authentic standards whenever they were available. Quantification of VOCs was conducted on an Agilent 7890B GC-FID equipped with an HP-1 column (30 m × 0.32 mm of inner diameter × 0.25 μm film thickness), using the same temperature programs in GC-MS analysis.

### Exposure of Citrus Plants to Synthetic Volatile Organic Compounds

The volatile dispensers (1.5 ml polyethylene flat bottom) were set according to Mérey et al. (2011) and Zhang et al. (2019). Synthetic (*E*)-β-ocimene, linalool, (*E*)-4,8-dimethyl-1,3,7-nonatriene (DMNT) and (*E*)-β-caryophyllene (purity ≥90%; TRC, Toronto, Canada; Sigma-Aldrich, Shanghai, China or Shanghai Yuanye Bio-Technology, Shanghai, China) were dissolved in dichloromethane at 10, 5, 10, and 100 μg μl^–1^, respectively. A 100-μl particular volatile solution was pipetted into the dispenser filled with 100 mg of glass wool. The vial bodies were wrapped with aluminum foil for heat protection and to avoid photodegradation. Using these, we obtained 0.80, 1.02, 1.37, and 1.30 times the release rates of these compounds from the guava odor source, respectively (Supplementary Figure 2 and Supplementary Table 4). In addition, two-component blends (DMNT and (*E*)-β-caryophyllene) and four-component blends (DMNT, (*E*)-β-caryophyllene, (*E*)-β-ocimene and linalool) were used. Plants exposed to methyl jasmonate (MeJA; purity ≥95%, Sigma-Aldrich, Shanghai, China) at a concentration of 5 μg μl^–1^ were used as the positive control. Dispensers that contained only 100 μl of dichloromethane were set as solvent controls. One single citrus plant and a volatile dispenser were placed in the glass chamber (Supplementary Figure 1B), during which charcoal-purified air was pumped into the system at 1 L⋅min^–1^. The dispensers were replaced every 3 days. After exposure for 14 days (volatiles) or 3 days (MeJA) with/without subsequent ACP infestation in the cages, insect preference, nymphal performance, and phytohormones were tested using the same criteria as above. The expression of defense genes (*LOX2_2* and *PI-like*) in citrus leaves that had been exposed for 3, 7, and 14 days or not exposed was also tested. Five replicates were used for each treatment.

### Gas Chromatography-Electroantennographic Detection Analysis

Coupled GC-EAD analysis was conducted on an Agilent 7890B GC equipped with an HP-1 column as explained earlier. The temperature program was the same as the one used for the GC-MS analysis. The column effluent was split in a ratio of 1:1 for simultaneous recording by an FID and EAD (Syntech, Hilversum, Netherlands). The entire antennae of the ACP female wasp were cut off, a reference electrode filled with ringer saline solution was connected to the base of the antenna, and the recording electrode was connected to the tip of the antenna (Wang et al., 2020). Analysis was conducted with headspace VOCs (2 μl) collected from guava VOC-exposed + ACP-infested citrus plants. Three replicates were conducted for every test.

### Olfactory Preference of Insects for Synthetic Volatile Organic Compounds

A total of 10 synthetic VOCs, including sabinene, myrcene, limonene, (*E*)-β-ocimene, linalool, β-elemene, (*E*)-β-caryophyllene, (*E*,*E*)-α-farnesene, DMNT, and (*E*,*E*)-4,8,12-trimethyl-1,3,7,11-tridecatetraene (TMTT) (all purity ≥90%; obtained from TRC, Toronto, Canada; Sigma-Aldrich, Shanghai, China or Shanghai Yuanye Bio-Technology, Shanghai, China), were dissolved in paraffin oil at concentrations of 0.01, 0.1, and 1 μg μl^–1^, respectively. The olfactory responses of insects to synthetic volatiles were evaluated using the Y-tube olfactometer as discussed earlier, with a minor modification. In brief, Y-tube arms with 200-ml min^–1^ airflow were connected to the 1.5-L glass jars containing 10 μl of a single volatile solution (total: 0.1, 1, or 10 μg, respectively) or paraffin oil (control). A single ACP or female wasp was released and observed within 8 min. Pooled responses of 120 ACP or 50 female wasps were obtained for each treatment.

### Statistical Analysis

Data were submitted to variance homogeneity and analyzed using ANOVA, and Tukey’s test was used to analyze significant differences among multiple comparisons. Student’s *t*-test and the chi-square (χ^2^) test were used to compare the two treatments. Usually, data with unequal variances will have normality issues as well (taking log or square root). PCA and heatmapping were conducted on MetaboAnalyst 5.0^1^.

## Results

### Exposure of Citrus Plants to Guava Volatile Organic Compounds Suppresses Asian Citrus Psyllid Preference and Performance

**FIGURE 1:**
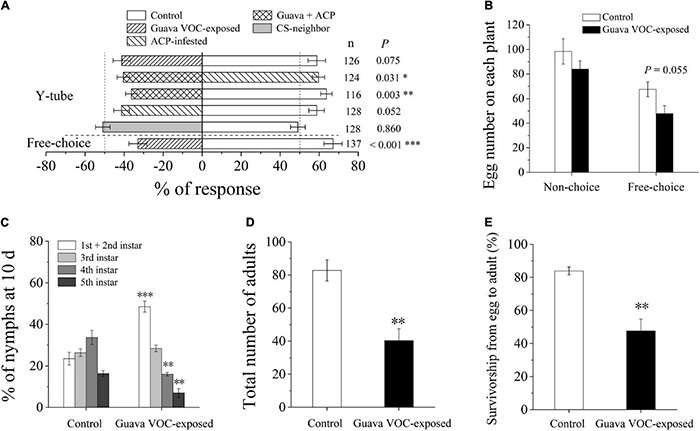
Exposure to guava volatile organic compounds (VOCs) enhances the resistance of citrus plants to Asian citrus psyllid (ACP). **(A)** Olfactory preference of ACP adults. Guava + ACP, guava VOC-exposed + ACP-infested. **(B)** Oviposition preference of ACP female wasps. **(C)** Performance of ACP nymphs at day 10. **(D)** The total number of ACP adults after emergence on each plant. **(E)** Survivorship from egg to adult. Data are mean ± SE (*n* = 5). Asterisks indicate statistical differences between exposure and control based on the χ^2^ test **(A)** and Student’s *t*-test **(B–E)** (*P < 0.05, ^**^P < 0.01, and ^***^P < 0.001). CS-neighbor, a citrus neighbor instead of guava neighbor; *n*, number of respondents.

Guava VOC-exposed citrus plants were avoided by ACP in the Y-tube and free choice behavioral experiments (Figure 1A). ACP also did not prefer citrus plants that were pre-infested with ACP. The number of eggs laid by female A on the guava VOC-exposed plants was reduced but did not show a significant difference compared with the control in the choice test (*P* = 0.055; Figure 1B). It was also unsuitable for hatched nymphs to grow on guava VOC-exposed citrus plants; in particular, the development of ACP was considerably negatively affected (Figure 1C), and the number of nymphs and adults was reduced (Figures 1D,E).

### Transcriptome Profiling of Citrus Plants After Exposure to Guava Volatile Organic Compounds

**FIGURE 2:**
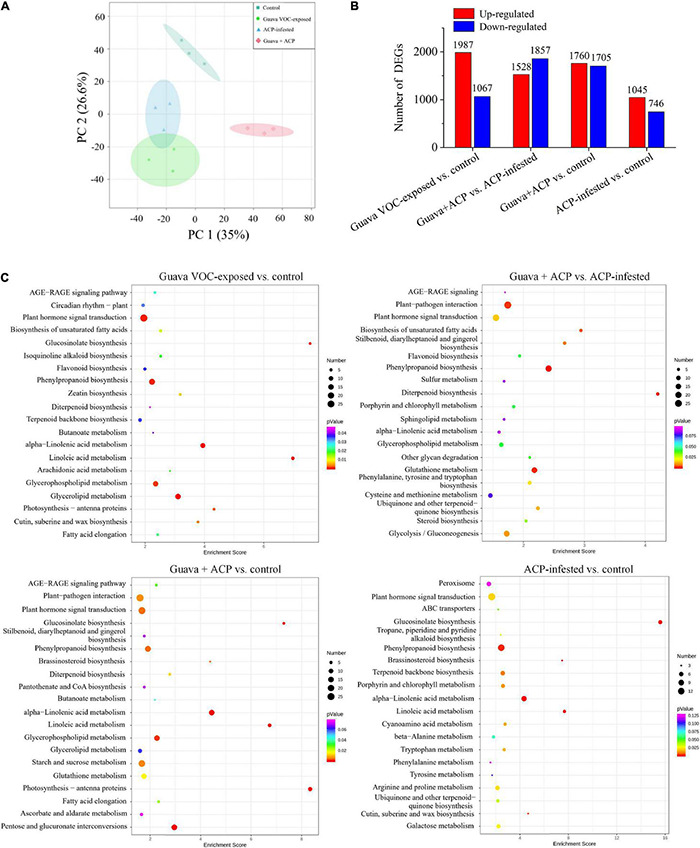
Effects of guava VOC exposure and ACP infestation on the transcriptome of citrus leaves. **(A)** Principal component analysis plots of transcript profiles. **(B)** The number of upregulated and downregulated differentially expressed genes (DEGs) in each comparison group. **(C)** KEGG enrichment (top 20) of DEGs.

Upon guava VOC exposure and ACP infestation, a series of genes were upregulated or downregulated (Figures 2A,B). Significantly enriched GO terms included the defense response, hormone-mediated signaling pathway, linoleate 13S-lipoxygenase activity, and terpenoid biosynthetic process (Supplementary Note 1). The KEGG pathway enrichment analysis revealed that the DEGs in transcripts were significantly enriched in plant defense-related pathways, such as plant hormone signal transduction, phenylpropanoid biosynthesis, alpha-linolenic acid metabolism, terpenoid backbone biosynthesis, diterpenoid biosynthesis, and flavonoid biosynthesis pathways (Figure 2C).

**FIGURE 3:**
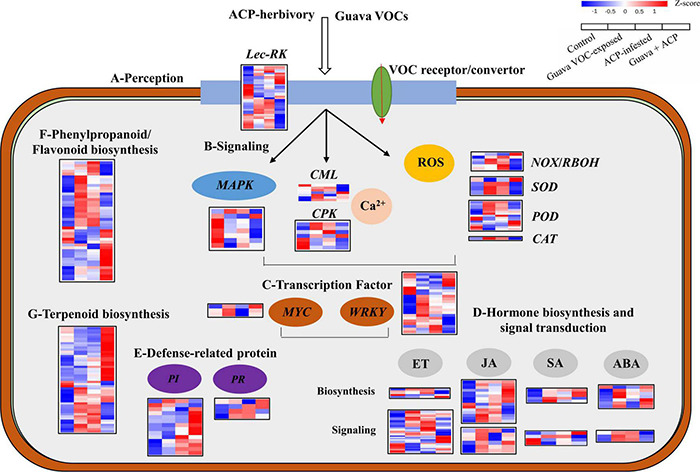
Expression profiles of DEGs in response to biological stimuli. **(A)** Perception, **(B)** signaling, **(C)** transcription factors, **(D)** hormones, **(E)** defense-related proteins, **(F,G)** secondary metabolism (phenylpropanoids, flavonoids, and terpenoids). See Supplementary Note 2 for details.

Multiple genes involved in cell communication, biosynthesis, and signaling were upregulated or downregulated (Figure 3). Genes responsible for the perception of insects and bacteria, such as those encoding *G-* and *L-type receptor kinase* (*Lec-RK*), were upregulated in guava VOC-exposed and ACP-infested citrus compared with the control. Early signaling events, including *calcium-binding protein* (*CML*) and *calcium-dependent protein kinase* (*CPK*), were differentially expressed. Meanwhile, most signal transduction-related genes, *mitogen-activated protein kinases* (*MAPKs*), were downregulated. *NADPH oxidase* (*NOX*), *respiratory burst oxidase homolog protein* (*RBOH*), *superoxide dismutase* (*SOD*), *peroxidase* (*POD*), and *catalase* (*CAT*), which are involved in the production and generation of reactive oxygen species (ROS), were upregulated. In the phenylpropanoid and flavonoid biosynthesis pathways, 15 upregulated and 5 downregulated DEGs were identified. A total of 49 DEGs involved in the terpenoid biosynthesis, including *AFAR-like*, *GGPPS7*, *GES*, and *CYP82G1-like*, which are related to the synthesis of α-farnesene, DMNT, and TMTT, were upregulated. A total of 42 DEGs were involved in phenylpropanoid and flavonoid biosynthesis. Five members of *pathogenesis-related genes* (*PRs*) were differentially expressed. In addition, among the 14 differentially expressed *PIs*, most *PI* genes were upregulated, especially in guava + ACP treatment. The expression of genes involved in ABA, ET, and SA biosynthesis and signaling was variably upregulated and downregulated. The expression of most JA biosynthetic pathway genes, including *LOX2*, *LOX6*, *AOC*, *OPR3*, and *OPR11*, was significantly increased, and the induction was stronger in guava + ACP-treated citrus. Among the biotic stress-related transcription factor genes, one *MYC2* gene was upregulated, whereas 22 members of the *WRKY* family genes were differentially expressed.

In the qRT-PCR analysis, the expression patterns of 14 genes involved in JA biosynthesis and signaling (*LOX2_2*, *OPR11_2*, *JMT-like*, and *MYC2-like*), SA biosynthesis (*PAL_2* and *PAL_3*), terpenoid biosynthesis (*GGPPS7*, *GES*, *CYP82G1-lik*e, and *AFAR-like*), phenylpropanoid biosynthesis (*COMT_2* and *PER4-like*) and direct defense-related genes (*lectin-like* and *PI-like*) were induced at different levels by guava VOC exposure and ACP infestation, which were generally in good agreement with the RNA-seq data (Supplementary Figure 3).

### Phytohormone Levels of Citrus Plants Increased After Exposure to Guava Volatile Organic Compounds

**FIGURE 4:**
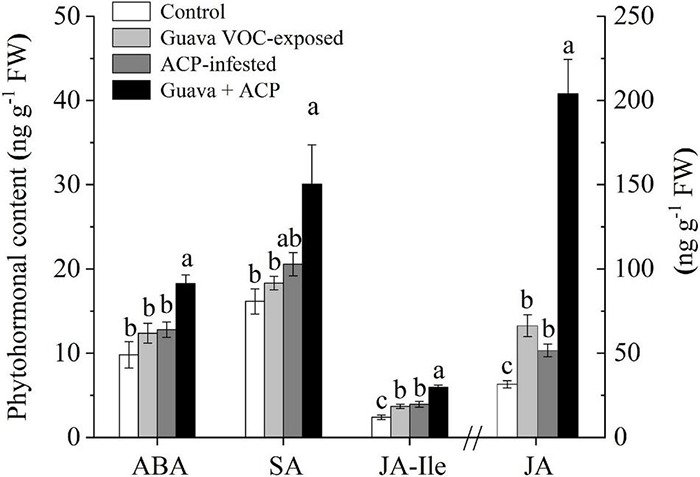
Phytohormone levels in citrus leaves that were exposed to guava VOCs and infested with ACP. Different letters on the columns (mean ± SE, *n* = 6) indicate significant differences based on Tukey’s multiple comparisons (*P* < 0.05).

The JA contents in guava VOC-exposed plants were significantly induced relative to the control (2.10-fold). ACP infestation also induced the accumulation of JA. The induction effect on JA levels was increased in exposed plants after ACP infestation compared with herbivory only (3.95-fold). JA-Ile levels showed an increasing tendency after exposure and infestation. Exposed plants that were not infested with ACP showed no differences in ABA and SA levels when compared with control plants, while the levels in exposed citrus plants that were infested with ACP increased (Figure 4).

### Semipolar and Volatile Specialized Metabolism of Citrus Plants After Exposure to Guava Volatile Organic Compounds

**FIGURE 5:**
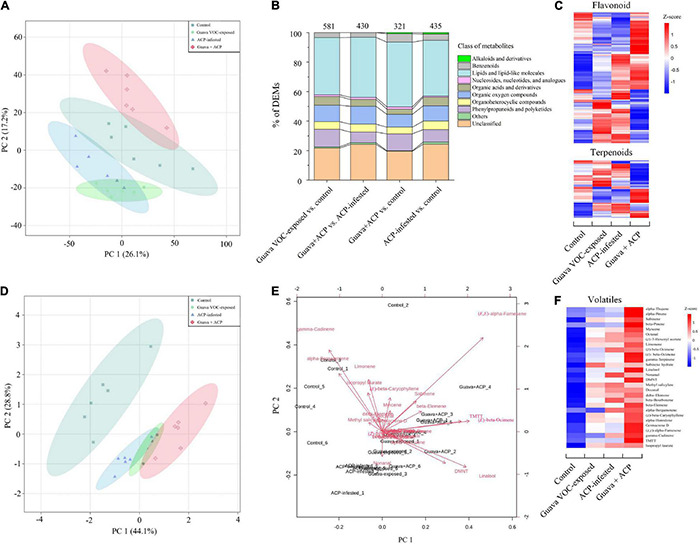
Differentially expressed metabolites (DEMs) in citrus leaves that were exposed to guava VOCs and infested with ACP. **(A)** PCA plots of semipolar metabolite profiles. **(B)** Percentages of DEMs in the four comparable groups (see Supplementary Table 2 for details). The total number of different metabolites is marked on the columns. **(C)** Heatmap of DEMs related to flavonoids and terpenoids (see Supplementary Note 4 for details). **(D,E)** PCA score plot and biplot of citrus leaf volatiles. **(F)** Heatmap of citrus volatile features (see Supplementary Table 3 for details).

Principal component analysis with all metabolite features showed a clear separation between different groups, suggesting that there were large metabolic differences between the plant response to guava VOC exposure/ACP infestation and the control (Figure 5A). Prenol lipids, phenylpropanoids and flavonoids, and organic oxygen compounds accounted for a higher proportion of the identified metabolites (Figure 5B). Meanwhile, the differentially expressed metabolites (DEMs), such as phenylpropanoids, flavonoids, and terpenoids, were more upregulated than downregulated in guava VOC-exposed, ACP-infested, and guava + ACP-treated citrus plants compared with the control (Figure 5C).

The GC-MS analysis of the volatiles collected from treatment and control citrus plants revealed differences in volatile profiles (Figure 5D). A total of 28 major compounds were consistently released, depending on the treatments (Figure 5F and Supplementary Table 3). Guava VOC exposure and ACP feeding resulted in considerably stronger induction of the release of VOCs by the citrus plants, and the combined treatment had the most dramatic effect and resulted in increases in the release of virtually all of the detected VOCs. The release of homoterpene DMNT and TMTT was greater in exposed plants. Loading analysis of PCA also showed that (*E*,*E*)-α-farnesene, linalool, (*E*)-β-ocimene, TMTT, and DMNT contributed more to the discrimination between treatments using the first two PCA components (Figure 5E).

### Identification of Active Guava Volatile Organic Compounds

In guava VOCs, sesquiterpenes were much more abundant than monoterpenes (Supplementary Table 4). Leaf tissue contained 24 kinds of volatile profiles, including high quantities of (*E*)-β-caryophyllene, DMNT, linalool, and (*E*)-β-ocimene. Except for (*E*)-β-caryophyllene, the major compounds released from roots contained 3-octanone, 1-nonanol, and an unknown volatile. For combining root and leaf VOCs, (*E*)-β-caryophyllene and DMNT were the most released VOCs in guava.

**FIGURE 6:**
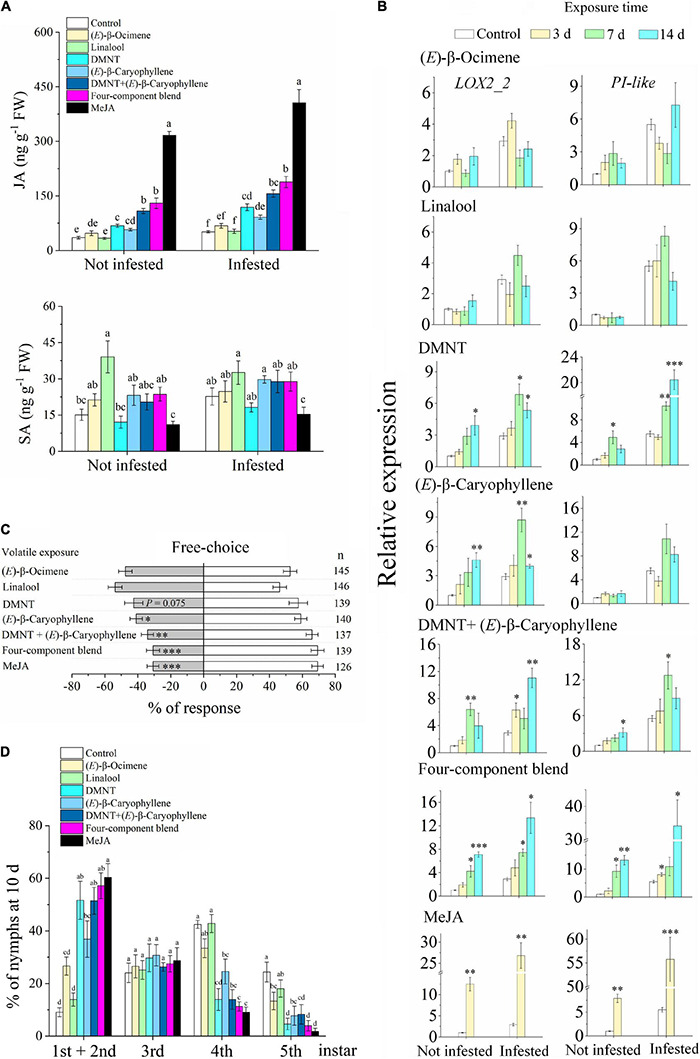
Exposure to synthetic VOCs activates citrus defense systems and suppresses ACP performance. **(A)** JA and SA levels (*n* = 5). **(B)** Expression of *LOX2_2* and *PI-like* (*n* = 3). **(C)** Behavioral preference of ACP in a free-choice experiment. Four-component blend, a blend of (*E*)-β-ocimene, linalool, DMNT, and (*E*)-β-caryophyllene. **(D)** Performance of ACP nymphs at day 10 (*n* = 5). Data showed as means ± SE. Significance was tested based on Tukey’s multiple comparisons **(A,D)** (different letters, *P* < 0.05), Student’s *t*-test **(B)**, and the χ^2^ test **(C)** (*P < 0.05, ^**^P < 0.01, and ^***^P < 0.001). *n*, number of respondents.

Exposure to DMNT, (*E*)-β-caryophyllene, the DMNT + (*E*)-β-caryophyllene blend, the four-VOC component blend, and MeJA increased the JA levels in the citrus plants compared with the control (Figure 6A). Exposure to single (*E*)-β-ocimene and linalool did not affect JA levels, while linalool (not infested) showed an increase in SA levels. (*E*)-β-ocimene and linalool did not affect the expression of *LOX2_2* (Figure 6B). DMNT increased the expression of *LOX2_2* and *PI-like* genes with or without subsequent ACP infestation. (*E*)-β-caryophyllene can induce the expression of *LOX2_2* but has no significant effect on *PI-like* expression. Meanwhile, exposure to MeJA and two- and four-VOC blends had a considerable effect on inducing the expression of these two genes.

The preference of ACP for citrus plants that were exposed to (*E*)-β-caryophyllene and two- and four-VOC blends was reduced compared with the control (Figure 6C). DMNT exposure also reduced the attraction to ACP, although it was not statistically significant (*P* = 0.075). The performance of ACP nymphs on citrus plants that had been exposed to DMNT, (*E*)-β-caryophyllene, and the blends was suppressed, with a higher percentage of low-instar nymphs than the control (Figure 6D). These results match the expected induction patterns and ACP preference based on the results of exposure to natural VOCs from guava.

### Electrophysiology and Olfactory Responses of Asian Citrus Psyllid to Citrus Volatile Organic Compounds

**FIGURE 7:**
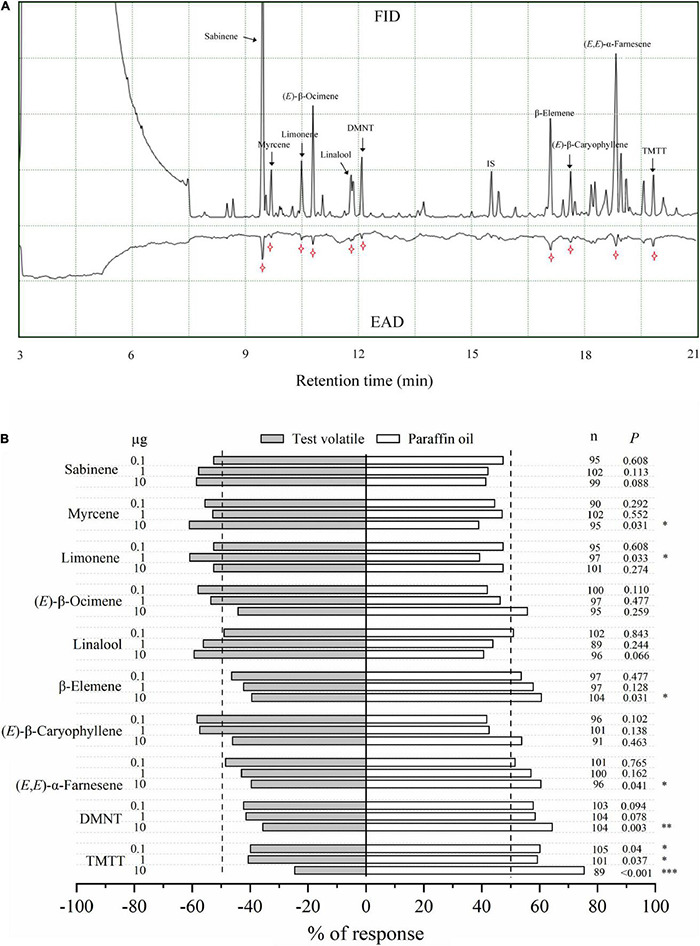
Electrophysiological and olfactory responses of ACP female wasps to individual VOCs. **(A)** GC/FID-EAD response of ACP female wasps to VOCs released from guava VOC-exposed citrus. The antennal potential activity was marked with a star symbol. **(B)** Olfactory response of ACP female wasps to different doses of synthetic VOCs in a Y-tube olfactometer. Asterisks indicate statistical differences based on the χ^2^ test (*P < 0.05, ^**^P < 0.01, and ^***^P < 0.001). *n*, number of respondents.

The electrophysiological activity of ten VOCs from guava VOC-exposed citrus was confirmed by antennal recordings (Figure 7A). A Y-tube behavioral experiment was also used to confirm the preference of ACP female adults for these compounds, which elicited high electrophysiological responses in ACP antennae. The results showed that myrcene and limonene had attraction effects on ACP adults compared with paraffin oil at 10 μg and 1 μg, respectively. However, β-elemene, (*E*,*E*)-α-farnesene, DMNT, and TMTT could repel ACP at certain concentrations (Figure 7B). Meanwhile, sabinene, (*E*)-β-ocimene, linalool, and (*E*)-β-caryophyllene (at 0.1, 1, and 10 μg) did not significantly affect the preference of ACP adults in the Y-tube, although these volatiles have an electrophysiological activity to ACP antennae.

### Exposure to Guava Volatile Organic Compounds Increases the Attractiveness of Citrus Plants to Parasitic Wasps

**FIGURE 8:**
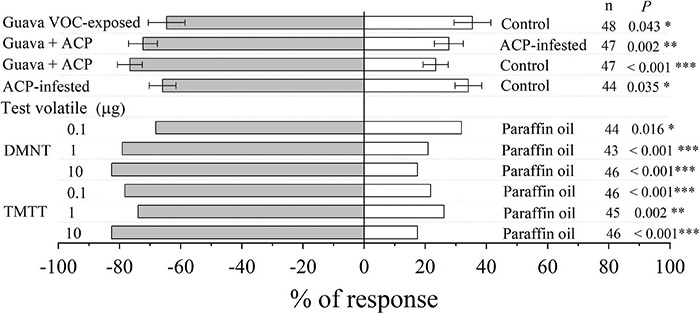
Olfactory response of parasitic female wasp (*T. radiata*) to citrus VOCs and synthetic VOCs in a Y-tube olfactometer. Asterisks indicate statistical differences based on the χ^2^ test (*P < 0.05, ^**^P < 0.01, and ^***^P < 0.001). *n*, number of respondents.

Compared with control plants, parasitic female wasps (*T. radiata*) were attracted to citrus plants that had been exposed to guava VOCs, and the attractiveness was stronger after infestation with ACP. In addition, female wasps were also significantly attracted by synthetic DMNT and TMTT (Figure 8).

## Discussion

Monoterpenes and sesquiterpenes serve various signaling functions in interspecific interactions (Ninkovic et al., 2019). In response to these VOC signals, a plant can exhibit a multitude of adaptation responses. Current results identified that guava VOCs were able to prime and induce defense responses involving early defense signaling and the production of defense proteins and metabolites in neighboring citrus plants, thereby increasing herbivore resistance.

The early defenses induced by VOCs are usually associated with Ca^2+^ signaling, MAPK signaling, WRKY regulators, and phytohormone biosynthesis (Ye et al., 2021). They respond rapidly to stimuli, leading to the activation of specific downstream targets to elicit the biosynthesis of defense-related signal molecules through synergistic or antagonistic interactions (Chen et al., 2020). As shown here, the expression of several genes involved in Ca^2+^ signaling and WRKYs was significantly induced with guava VOC exposure and ACP infestation. JA outbreak is an important mechanism of VOC-induced plant resistance. VOCs can cause plants to enter a “priming” state, resulting in a stronger and/or faster defense response, and the “priming” effect is closely related to JA signaling (Mauch-Mani et al., 2017). 13-Lipoxygenases (13-LOXs) are involved in wound-induced JA formation with a partially specific activity in which LOX2 plays a dominant role in wounding and lipid peroxidation (Wasternack and Song, 2017). *MYC2*, which is well known as the master regulator of JA signaling, can positively regulate the expression of *LOX* (Lorenzo et al., 2004). In this study, the expression levels of a variety of JA biosynthesis and signaling pathways (*LOX2*, *LOX6*, *AOC, OPR*, and *MYC2-like*) and the production of JA and JA-Ile increased simultaneously in citrus plants that were exposed to guava VOCs. Exposure to (*E*)-β-caryophyllene and DMNT, the main components of guava VOCs, also induced the expression of *LOX2* and/or *PI-like* and the accumulation of JA in citrus leaves. (*E*)-β-Caryophyllene can bind to the TOPLESS-like (TPL-like) proteins, leading to TPL release and then activation of *MYC2* in JA signaling (Nagashima et al., 2019). In addition, DMNT can directly increase cytoplasmic Ca^2+^ influx and induce defense by upregulating the expression of *PI* and *LOX* and inducing the accumulation of JA (Asai et al., 2009; Chen et al., 2021; Jing et al., 2021). Therefore, we hypothesize that guava VOCs alter ion permeability and Ca^2+^ influx across the plasma membrane after their binding to the cell membrane or VOC receptors/convertors in receiver citrus plants. This possibly triggers oxidative burst, transcription factor, and phytohormone signaling, thereby boosting defense responses, as chemical elicitors resulting from herbivory and pathogenesis are known to do (Asai et al., 2009; Sukegawa et al., 2018).

Notably, exposure to mixed volatile cues showed a stronger defense response in JA-related signaling compared with exposure to individuals, despite their limited role in JA induction by a single (*E*)-β-ocimene or linalool at current concentrations. Plants modulate the strength of their responses based on the reliability of detected VOC cues, while the integration of multiple VOC cues, combined with crosstalk of plant internal signaling, is often more reliable and robust than a single VOC signal (Šimpraga et al., 2016; Frank et al., 2021). In guava and citrus interactions, DMNT and (*E*)-β-caryophyllene should be part of a more complex VOC blend forming signals with other compounds, especially (*E*)-β-ocimene, linalool, α-humulene (α-caryophyllene), and (*E*)-nerolidol, which has been found to play an important role in the plant–plant communication (Kang et al., 2018; Nagashima et al., 2019; Zhang et al., 2019; Chen et al., 2020). These results match the expected induction patterns based on the results of exposure to constructively released VOCs from guava, emphasizing the importance of DMNT and (*E*)-β-caryophyllene in interspecific interactions.

The reprogramming of metabolic pathways by JA ultimately triggers the secretion of plant secondary metabolites. Flavonoids are an important group of phenolic compounds produced directly from the phenylpropanoid pathway (Vogt, 2010). Their presence can alter the palatability of plants, reduce their nutritional value, decrease digestibility, and even act as toxins (Mierziak et al., 2014), which are associated with defense responses in plants against insect herbivores, such as moths, mites, and whiteflies (Mallikarjuna et al., 2004; Mierziak et al., 2014; Zhang et al., 2020; Xia et al., 2021). Liu et al. (2020) also revealed that a series of metabolites in the biosynthesis pathways of phenylpropanoids and flavonoids were involved in responding to ACP infestation. High levels of flavonoids can inhibit insect feeding or directly delay the growth and development of nymphs, thereby increasing mortality (Xia et al., 2021). This may account for the significant increase in the percentage of low-instar nymphs on guava VOC-exposed citrus, which is consistent with that of MeJA exposure.

Volatile terpenoids are the major products of plant VOCs. The diversity of terpenes is expanded as a result of terpene synthases (TPSs), which can use different phenyl diphosphates as substrates. The expression of citrus genes related to terpenoid synthesis (*AFAR-like*, *GGPPS7*, *GES*, and *CYP82G1-like*) and the emission of terpenes (especially DMNT and TMTT) were upregulated after exposure to guava VOCs. The P450 enzyme CYP82G1 can convert both (*E*,*E*)-geranyllinalool and (*E*)-nerolidol to TMTT and DMNT, respectively (Lee et al., 2010; Brillada et al., 2013). It seems that quantitative and/or qualitative changes in VOC emissions can negatively or positively affect the host- or prey-seeking behavior of insects, as it has been shown that insects can sense even small amounts of VOCs (Bruce et al., 2005; Salvagnin et al., 2018). Behavioral and electroantennographic studies demonstrated that β-elemene, (*E*,*E*)-α-farnesene, DMNT, and TMTT can repel ACP at a certain concentration. In addition, DMNT and TMTT are more attractive to the parasitic wasp *T. radiata*. Thus, the change in volatile profiles caused by interactions between undamaged plants may be a mechanism contributing to these observations.

**FIGURE 9:**
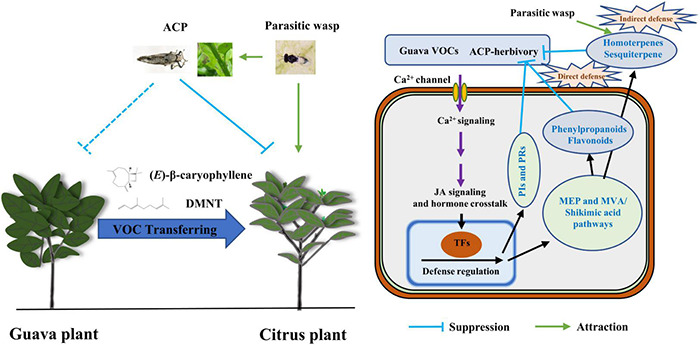
A working model of guava VOCs that triggers direct and indirect defense responses to ACP in neighboring citrus plants. Insects are not drawn to scale.

In summary, this study revealed a novel, ecologically relevant function of guava VOCs in the context of the “volatile language” of plants (Figure 9). Volatile terpenoids, which are also constitutively released by guava, are important substances for interactions between multi-trophic levels. Our study indicated that volatile signals from undamaged guava plants mediate similar effects on tri-trophic interactions as signals from herbivore-attacked plants. Thus, guava VOCs should play a synergistic role in pest management because guava VOCs can not only repel insect herbivores and attract herbivore enemies but can also boost JA-dependent anti-herbivore activities in citrus plants. These results add to the understanding of the mechanism of volatile communications in coexisting and competitive plants and also provide new insight into the roles of guava VOCs in the ecological management of herbivore pests in intercropping systems. In addition, rhizosphere volatiles and exudates, as well as microbes underground, are implicated in plant–plant interactions (Ninkovic et al., 2021; Sharifi and Ryu, 2021; Wang et al., 2021). As the experimental plants were in pots and there was no connection through the rhizosphere, volatiles rather than root exudates and the microbiome were implicated in the plant–plant interaction observed in this study. The potential ecological significance of microbes and rhizosphere chemistry in guava–citrus interactions should be elucidated in the future.

## Data Availability Statement

The datasets presented in this study can be found in online repositories. The names of the repository/repositories and accession number(s) can be found in the article/Supplementary Material.

## Author Contributions

XZ, JL, and SL conceived and designed the experiments. SL, SR, and TX performed the experiments and analyzed the data. YG and SW conducted the GC-MS and LC-MS measurements. SL and XZ wrote the manuscript. All authors contributed to the article and approved the submitted version.

## Conflict of Interest

The authors declare that the research was conducted in the absence of any commercial or financial relationships that could be construed as a potential conflict of interest.

## Publisher’s Note

All claims expressed in this article are solely those of the authors and do not necessarily represent those of their affiliated organizations, or those of the publisher, the editors and the reviewers. Any product that may be evaluated in this article, or claim that may be made by its manufacturer, is not guaranteed or endorsed by the publisher.

## Footnotes

1
https://www.metaboanalyst.ca
