# Supplementary material for: Volatile Signals From Guava Plants Prime Defense Signaling and Increase Jasmonate-Dependent Herbivore Resistance in Neighboring Citrus Plants
Source: Front Plant Sci. 2022 Mar 10;13:833562. doi: 10.3389/fpls.2022.833562 (PMC8965645; doi:10.3389/fpls.2022.833562)
Supplement: Supplementary file 1 [file Data_Sheet_1.DOCX]

**Supplementary Tables:**

**Table S1** Gene-specific primer pairs for qRT-PCR.

| Gene name | Forward primer (5’-3’) | Reverse primer (5’-3’) | Product (bp) |
| --- | --- | --- | --- |
| *LOX2_2* | ATCCTAGACGTTGCAGAACTGG | TACCAACGCATGCAACACTG | 161 |
| *OPR11_2* | TGAAGCAACTGGGGTCTCTG | GGAGCTTCTCCATTTGGCTGA | 189 |
| *JMT-like* | ACATGCCAGATGTCGCAATC | TGAAGAACGCTGGCAATGTC | 113 |
| *MYC2-like* | AAGCACAATCGCATGGCATC | ACAGAGACTTGACTTGGTGGAC | 123 |
| *PAL_2* | CAAGTTTGTTGACGGGCGAA | AGAGGAGCACCATTCCACTC | 130 |
| *PAL_3* | CGTCGGTGCTAGTGGAGAG | TTCAGTGCGTGCTCAACAAG | 162 |
| *GGPPS7* | GCGGAGTGATAATCGGAGGAG | CCATCAGCTTTGGATACGTGG | 186 |
| *GES* | TGATTACCAATCGGTCGCCAA | TGAATCTAATTTTCGGCGGCA | 140 |
| *AFAR-like* | TGCAGTGGGGCTAGTTTATGA | ATCCCACCTTTCAACAGCAC | 154 |
| *CYP82G1-like* | AAGTCACCGGCTTGAATTGC | ACCTTCGCAGGATTGAATGC | 108 |
| *COMT_2* | ATGGCTTGACACCAGTTTCC | AGCCGCAAATCCATCCATTC | 182 |
| *PER4-like* | TCGGTACTGTTGGATGATACGG | TCTGCACACGAAACAACACG | 143 |
| *Lectin-like* | ATCAGACCCTTCACCAGAATCG | ACAAACGTCATGCCATCTGC | 168 |
| *PI-like* | TGGCCACAAAAGCTCAGTTG | AACGGACACTCTCCACTTGTG | 160 |
| *FBOX ** | TTGGAAACTCTTTCGCCACT | CAGCAACAAAATACCCGTCT | 112 |
| *UPL7 ** | CAAAGAAGTGCAGCGAGAGA | TCAGGAACAGCAAAAGCAAG | 150 |

***** *FBOX* and *UPL7* are used as reference genes.

**Table S2** Overview of DEMs in the four comparison groups, related to Figure 5B.

| Class | Guava VOC-exposed vs. control | | | Guava + ACP vs. ACP-infested | | | Guava + ACP vs. control | | | ACP-infested vs. control | | |
| --- | --- | --- | --- | --- | --- | --- | --- | --- | --- | --- | --- | --- |
|  | Up | Down | Total | Up | Down | Total | Up | Down | Total | Up | Down | Total |
| Benzenoids | 4 | 10 | 14 | 13 | 7 | 20 | 5 | 8 | 13 | 5 | 14 | 19 |
| Lipids | 74 | 55 | 129 | 77 | 83 | 160 | 101 | 37 | 138 | 100 | 22 | 122 |
| Prenol lipids (terpenoids) | 12 | 6 | 18 | 14 | 16 | 30 | 10 | 13 | 23 | 9 | 3 | 12 |
| Organic acids and derivatives | 8 | 12 | 20 | 16 | 18 | 34 | 9 | 9 | 18 | 12 | 13 | 25 |
| Organic oxygen compounds | 28 | 24 | 52 | 45 | 20 | 65 | 21 | 9 | 30 | 19 | 26 | 45 |
| Organoheterocyclic compounds | 10 | 13 | 23 | 17 | 13 | 30 | 13 | 14 | 27 | 12 | 13 | 25 |
| Phenylpropanoids and Flavonoids | 34 | 16 | 50 | 44 | 54 | 98 | 49 | 22 | 71 | 48 | 20 | 68 |
| Others | 3 | 8 | 11 | 8 | 4 | 12 | 3 | 2 | 5 | 5 | 9 | 14 |
| Unclassified | 62 | 39 | 101 | 48 | 76 | 124 | 62 | 46 | 108 | 77 | 30 | 107 |
| Total number | 235 | 183 | 418 | 282 | 291 | 573 | 273 | 160 | 433 | 287 | 150 | 437 |

**Table S3** Volatile emissions from citrus plants after exposure to guava VOCs and ACP infestation.

| Peak No. | Volatile name ^a^ | RT ^b^ | RI ^b^ | ng h^-1^ g^-1^ DW (n = 6) ^d^ | | | |
| --- | --- | --- | --- | --- | --- | --- | --- |
|  |  |  |  | Control | Guava VOC-exposed | ACP-infested | Guava + ACP |
| 1 | α-Thujene ^A, M^ | 6.71 | 928 | tr. ^c^ | 0.21 ± 0.02 b | 0.35 ± 0.05 b | 1.00 ± 0.11 a |
| 2 | α-Pinene ^B, M^ | 6.86 | 936 | tr. | 0.20 ± 0.02 b | 0.26 ± 0.02 b | 1.05 ± 0.06 a |
| 3 | Sabinene ^B, M^ | 7.64 | 976 | 13.84 ± 1.56 c | 62.87 ± 6.44 b | 65.38 ± 4.97 b | 95.60 ± 3.46 a |
| 4 | β-Pinene ^B, M^ | 7.71 | 979 | tr. | 0.28 ± 0.03 b | 0.51 ± 0.07 b | 3.13 ± 0.37 a |
| 5 | Myrcene ^B, M^ | 7.95 | 992 | 2.10 ± 0.22 b | 8.55 ± 0.52 a | 10.34 ± 1.10 a | 14.00 ± 2.51 a |
| 6 | 1-Octanal ^A^ | 8.18 | 1004 | 0.31 ± 0.03 b | 1.42 ± 0.26 ab | 2.13 ± 0.39 a | 2.36 ± 0.38 a |
| 7 | (*Z*)-3-Hexenyl acetate ^B, GLV^ | 8.25 | 1007 | 0.28 ± 0.01 b | 2.24 ± 0.35 a | 2.72 ± 0.24 a | 2.87 ± 0.55 a |
| 8 | Limonene ^B, M^ | 8.71 | 1031 | 4.30 ± 0.45 c | 10.46 ± 0.69 b | 11.64 ± 0.63 b | 15.05 ± 0.97 a |
| 9 | (*Z*)-β-Ocimene ^B, M^ | 8.86 | 1038 | 0.25 ± 0.04 c | 1.06 ± 0.16 b | 2.07 ± 0.16 a | 1.97 ± 0.17 a |
| 10 | (*E*)-β-Ocimene ^B, M^ | 9.07 | 1049 | 2.39 ± 0.30 c | 16.74 ± 1.35 b | 21.23 ± 2.12 b | 46.19 ± 3.24 a |
| 11 | γ-Terpinene ^A, M^ | 9.30 | 1049 | tr. | 1.07 ± 0.12 b | 1.29 ± 0.19 b | 2.56 ± 0.22 a |
| 12 | Sabinene hydrate ^A, M^ | 9.49 | 1061 | 0.28 ± 0.02 b | 2.94 ± 0.20 a | 2.50 ± 0.23 a | 2.71 ± 0.23 a |
| 13 | Linalool ^B, M^ | 10.09 | 1102 | 0.35 ± 0.04 c | 9.11 ± 1.10 b | 10.41 ± 1.31 b | 25.08 ± 2.63 a |
| 14 | 1-Nonanal ^A^ | 10.15 | 1105 | 0.98 ± 0.18 c | 5.42 ± 0.53 b | 10.72 ± 1.33 a | 8.10 ± 1.57 ab |
| 15 | DMNT ^B, Ht^ | 10.38 | 1117 | 0.76 ± 0.23 c | 10.10 ± 1.45 b | 10.96 ± 0.78 b | 22.20 ± 2.96 a |
| 16 | Methyl salicylate ^B^ | 11.92 | 1199 | 0.98 ± 0.16 b | 3.13 ± 0.35 a | 4.02 ± 0.64 a | 3.20 ± 0.45 a |
| 17 | 1-Decanal ^A^ | 12.04 | 1207 | 0.82 ± 0.12 b | 2.89 ± 0.41 a | 3.82 ± 0.44 a | 3.80 ± 0.68 a |
| 18 | δ-Elemene ^A, S^ | 14.44 | 1345 | 0.88 ± 0.18 b | 2.88 ± 0.38 a | 3.44 ± 0.35 a | 3.96 ± 0.39 a |
| 19 | β-Bourbonene ^A, S^ | 15.28 | 1396 | 0.33 ± 0.03 c | 1.92 ± 0.25 b | 3.57 ± 0.43 a | 2.88 ± 0.37 ab |
| 20 | β-Elemene ^B, S^ | 15.35 | 1401 | 5.05 ± 0.77 c | 26.06 ± 2.07 b | 25.38 ± 3.35 b | 43.13 ± 5.00 a |

**Continued Table S3**

| Peak No. | Volatile name ^a^ | RT ^b^ | RI ^b^ | ng h^-1^ g^-1^ DW (n = 6) ^d^ | | | |
| --- | --- | --- | --- | --- | --- | --- | --- |
|  |  |  |  | Control | Guava VOC-exposed | ACP-infested | Guava + ACP |
| 21 | α-Bergamotene ^A, S^ | 15.71 | 1423 | 3.51 ± 0.48 a | 2.52 ± 0.21 a | 7.07 ± 1.35 a | 6.88 ± 0.75 a |
| 22 | (*E*)-β-Caryophyllene ^B, S^ | 15.86 | 1433 | 3.00 ± 0.24 b | 11.32 ± 1.83 a | 11.03 ± 1.01 a | 16.52 ± 2.17 a |
| 23 | α-Humulene ^B, S^ | 16.41 | 1467 | 0.35 ± 0.04 b | 3.49 ± 0.63 a | 3.58 ± 0.42 a | 5.45 ± 0.82 a |
| 24 | Germacrene D ^A, S^ | 16.83 | 1494 | 1.45 ± 0.31 c | 5.15 ± 0.39 b | 4.91 ± 0.55 b | 10.16 ± 0.57 a |
| 25 | (*E*,*E*)-α-Farnesene ^B, S^ | 17.12 | 1513 | 14.75 ± 1.67 c | 63.87 ± 6.02 b | 63.15 ± 4.86 b | 135.16 ± 3.98 a |
| 26 | γ-Cadinene ^A, S^ | 17.33 | 1527 | 8.32 ± 1.25 b | 9.09 ± 0.79 b | 17.39 ± 1.17 a | 21.49 ± 1.68 a |
| 27 | TMTT ^B, Ht^ | 18.17 | 1584 | tr. | 9.81 ± 1.09 b | 10.33 ± 0.96 b | 35.57 ± 1.27 a |
| 28 | Isopropyl laurate ^A^ | 18.82 | 1629 | 1.39 ± 0.16 b | 2.58 ± 0.19 a | 3.15 ± 0.28 a | 3.11 ± 0.09 a |
| GLVs | | | | 0.28 ± 0.01 b | 2.24 ± 0.35 a | 2.72 ± 0.24 a | 2.87 ± 0.55 a |
| Monoterpenes | | | | 23.53 ± 1.71 c | 113.49 ± 8.83 b | 125.97 ± 9.18 b | 208.35 ± 6.27 a |
| Sesquiterpenes | | | | 37.62 ± 2.82 c | 126.31 ± 7.60 b | 139.52 ± 2.57 b | 245.63 ± 8.98 a |
| Homoterpenes | | | | 0.76 ± 0.23 c | 19.91 ± 2.51b | 21.30 ± 1.07 b | 57.77 ± 2.74 a |
| Other volatiles | | | | 4.48 ± 0.30 c | 15.43 ± 1.50 b | 23.84 ± 2.61 a | 20.57 ± 2.77 ab |
| Total | | | | 66.67 ± 3.32 c | 277.38 ± 17.67 b | 313.34 ± 14.36 b | 535.20 ± 15.67 a |

^a^ A = Identification by comparison of mass spectrum and retention index according to NIST library and literature; B = Identification by comparison of mass spectrum and retention index according to authentic standard; M = monoterpenes, S = sesquiterpenes, Ht = homoterpenes, DMNT = (*E*)-4,8-dimethyl-1,3,7-nonatriene, TMTT = (*E*,*E*)-4,8,12-trimethyl-1,3,7,11-tridecatetraene; DW = dry weight.

^b^ RT = retention time, RI = retention index obtained experimentally (calculated) on Agilent 8890 GC-5977B MSD.

^c^ tr. = trace level, < 0.01%.

^d^ Guava-exposed, citrus plants were exposed to guava VOCs for14 d; ACP-infested, citrus plants were infested with ACP for 24 h; Guava + ACP, citrus plants were both exposure and infestation. Different letters on the rows (means ± SE) indicate significant differences by analysis of variance followed by Tukey's multiple comparison (*P* < 0.05), based on the emission of ng h^-1^ g^-1^ DW.

**Table S4** Identification and quantification of guava leaf and root VOCs.

| Peak No. | Volatile name ^a^ | RT ^b^ | RI ^b^ | ng h^-1^ g^-1^ DW (n = 6) | |  | ng h^-1^ plant^-1^ (n = 6) |
| --- | --- | --- | --- | --- | --- | --- | --- |
|  |  |  |  | Leaf ^c^ | Root ^d^ |  | Total emission ^e^ |
| 1 | Methyl hexanoate ^A^ | 6.54 | 924 | tr. | n.d. |  | tr. |
| 2 | α-Pinene ^B, M^ | 6.74 | 934 | tr. | 2.06 ± 0.44 |  | 7.14 ± 1.31 |
| 3 | Myrcene ^B, M^ | 7.84 | 991 | 0.97 ± 0.28 | n.d. |  | 17.60 ± 5.19 |
| 4 | 3-Octanone ^A^ | 7.88 | 993 | n.d. | 16.27 ± 1.14 |  | 157.63 ± 3.85 |
| 5 | Limonene ^B, M^ | 8.61 | 1030 | 2.62 ± 0.35 | 3.11 ± 0.16 |  | 57.81 ± 5.71 |
| 6 | (*Z*)-β-Ocimene ^B, M^ | 8.76 | 1038 | tr. | n.d. |  | tr. |
| 7 | (*E*)-β-Ocimene ^B, M^ | 8.97 | 1049 | 31.46 ± 2.52 | n.d. |  | 570.84 ± 58.47 |
| 8 | Linalool ^B, M^ | 9.98 | 1101 | 13.42 ± 1.81 | 2.05 ± 0.21 |  | 246.27 ± 28.62 |
| 9 | DMNT ^B, Ht^ | 10.28 | 1117 | 67.84 ± 4.73 | n.d. |  | 1221.22 ± 84.43 |
| 10 | 1-Nonanol ^A^ | 11.44 | 1179 | n.d. | 55.64 ± 4.07 |  | 195.89 ± 9.05 |
| 11 | α-Copaene ^A, S^ | 15.00 | 1384 | 13.80 ± 1.59 | 2.15 ± 0.19 |  | 257.83 ± 33.02 |
| 12 | 1-Tetradecane ^A^ | 15.24 | 1400 | 2.86 ± 0.33 | tr. |  | 51.71 ± 6.68 |
| 13 | α-Gurjunene ^A, S^ | 15.57 | 1421 | 1.56 ± 0.36 | n.d. |  | 27.47 ± 6.68 |
| 14 | (*E*)-β-caryophyllene ^B, S^ | 15.75 | 1432 | 305.00 ± 17.29 | 178.12 ± 18.72 |  | 6114.70 ± 307.09 |
| 15 | Aromandendrene ^A, S^ | 16.05 | 1451 | 12.12 ± 1.48 | n.d. |  | 219.51 ± 30.15 |
| 16 | α-Humulene ^B, S^ | 16.29 | 1466 | 5.85 ± 0.64 | 7.96 ± 0.54 |  | 134.69 ± 12.04 |
| 17 | γ-Muurolene ^A, S^ | 16.61 | 1486 | 13.31 ± 1.07 | n.d. |  | 240.30 ± 22.51 |
| 18 | α-Bisabolene ^A, S^ | 16.94 | 1507 | 8.16 ± 1.20 | 3.55 ± 0.17 |  | 160.08 ± 23.17 |
| 19 | β-Bisabolene ^A, S^ | 17.05 | 1515 | 13.53 ± 1.30 | n.d. |  | 244.01 ± 25.27 |
| 20 | γ-Cadinene ^A, S^ | 17.19 | 1524 | 2.95 ± 0.31 | 4.78 ± 0.33 |  | 70.00 ± 6.35 |

**Continued Table S4**

| Peak No. | Volatile name ^a^ | RT ^b^ | RI ^b^ | ng h^-1^ g^-1^ DW (n = 6) | |  | ng h^-1^ plant^-1^ (n = 6) |
| --- | --- | --- | --- | --- | --- | --- | --- |
|  |  |  |  | Leaf ^c^ | Root ^d^ |  | Total emission ^e^ |
| 21 | δ-Cadinene ^A, S^ | 17.32 | 1533 | 10.66 ± 2.05 | 3.17 ± 0.12 |  | 200.11 ± 37.59 |
| 22 | Cada-1,4-diene ^A, S^ | 17.46 | 1542 | 8.08 ± 0.48 | 3.28 ± 0.21 |  | 158.35 ± 12.47 |
| 23 | (*E*)-Nerolidol ^B, S^ | 17.85 | 1568 | 1.16 ± 0.42 | n.d. |  | 21.12 ± 7.53 |
| 24 | TMTT ^B, Ht^ | 18.05 | 1582 | 3.77 ± 2.56 | n.d. |  | 68.86 ± 10.17 |
| 25 | 1-Hexadecane ^A^ | 18.31 | 1600 | 6.31 ± 0.31 | tr. |  | 113.96 ± 7.81 |
| 26 | Unknown 1 | 18.46 | 1610 | n.d. | 23.92 ± 1.52 |  | 85.31 ± 6.68 |
| 27 | 1-Heptadecane ^A^ | 19.75 | 1701 | 7.03 ± 0.87 | tr. |  | 127.49 ± 17.54 |
| Monoterpenes | | | | 48.47 ± 3.03 | 7.23 ± 0.72 |  | 899.66 ± 61.50 |
| Sesquiterpenes | | | | 396.18 ± 24.33 | 203.02 ± 19.18 |  | 7723.38 ± 499.09 |
| Homoterpenes | | | | 71.61 ± 4.49 | n.d. |  | 1290.08 ± 90.26 |
| Other volatiles | | | | 16.20 ± 1.41 | 95.82 ± 4.11 |  | 631.99 ± 29.92 |
| Total emission | | | | 532.47 ± 30.03 | 306.07 ± 20.55 |  | 10545.12 ± 628.58 |
| Leaf/root dry weight (g) | | | | 18.04 ± 0.73 | 3.57 ± 0.19 |  | - |

^a^ A = Identification by comparison of mass spectrum and retention index according to NIST library and literature. B = Identification by comparison of mass spectrum and retention index according to authentic standard. M = monoterpenes, S = sesquiterpenes, Ht = homoterpenes, DMNT = (*E*)-4,8-dimethyl-1,3,7-nonatriene, TMTT = (*E*,*E*)-4,8,12-trimethyl-1,3,7,11-tridecatetraene; DW = dry weight.

^b^ RT = retention time, RI = retention index obtained experimentally (calculated) on Agilent 7890B GC-5977A MSD.

^c^ n.d. = not detection; tr. = trace level, < 0.01%.

^d^ Root VOVs has been subtracted from background VOCs of soil.

^e^ Total emission (means ± SE) is quantified in ng h^-1^ plant^-1^ based on the emission rate (ng h^-1^ g^-1^ DW) and dry biomass (g) of leaf and root.
