# Supplementary material for: Volatile Signals From Guava Plants Prime Defense Signaling and Increase Jasmonate-Dependent Herbivore Resistance in Neighboring Citrus Plants
Source: Front Plant Sci. 2022 Mar 10;13:833562. doi: 10.3389/fpls.2022.833562 (PMC8965645; doi:10.3389/fpls.2022.833562)
Supplement: Supplementary file 2 [file Data_Sheet_2.DOCX]

**Supplementary Figures：**

**
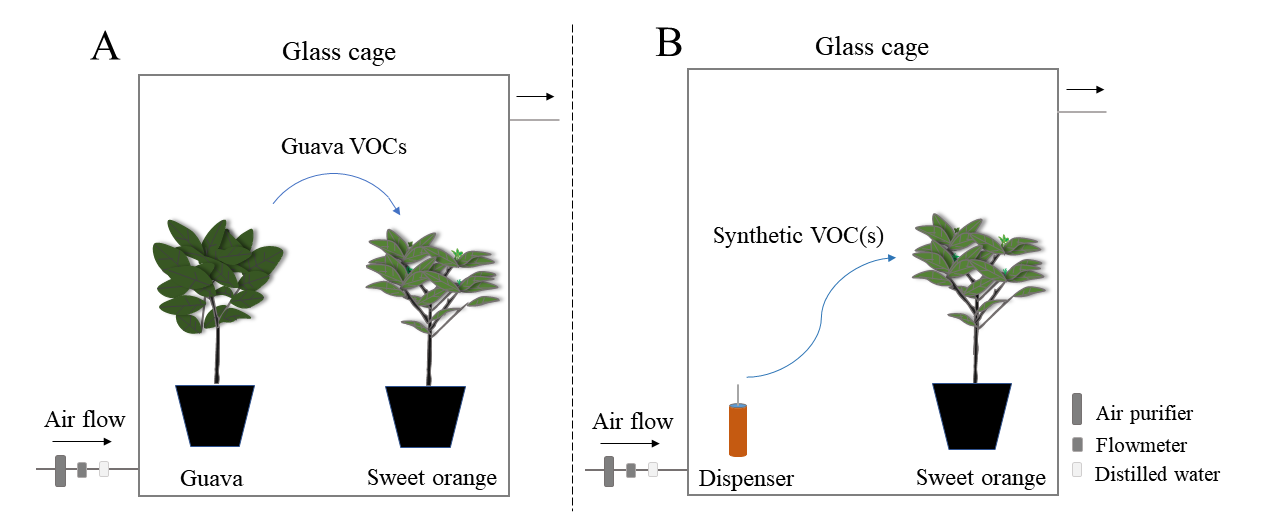
**

**Figure S1** Experimental setup for exposure of citrus plants to guava VOCs and synthetic VOCs.





**Figure S2** Emissions of (*E*)-β-ocimene, linalool, DMNT, and (*E*)-β-caryophyllene from guava plants and volatile dispensers. The release rate (mean ± SE, n = 6 or 4) of volatiles from the dispensers was quantified by GC at the second day. The emissions of guava volatiles are shown in Table S4.


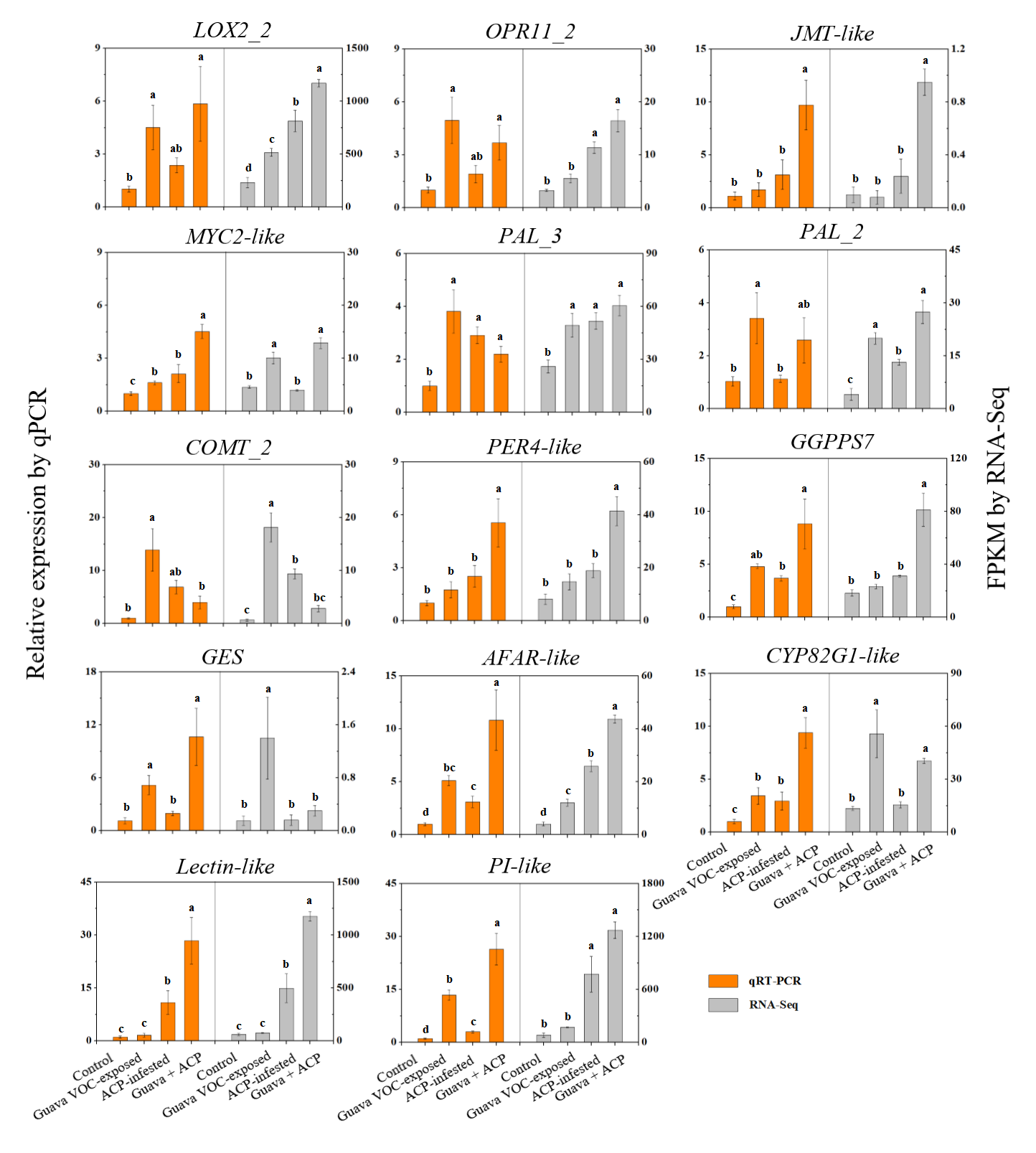


**Figure S3** Validation of the relative expression levels of candidate genes by qRT−PCR. Gene annotations are described in Note S3. Error bars correspond to SEs (n = 3). Different letters on the columns indicate significant differences among treatments from Tukey's multiple comparison (*P* < 0.05).
